# Use of Patient-Specific Titanium Plates to Prevent Iatrogenic Mandibular Fracture During the Surgical Removal of Dentigerous Cysts: A Two-Case Series

**DOI:** 10.7759/cureus.64520

**Published:** 2024-07-14

**Authors:** Hugo Bazin, Rufino Felizardo, Geraldine Lescaille, Juliette Rochefort, Soufiane Boussouni

**Affiliations:** 1 UFR Odontology, Faculté de Santé, Université Paris Cité, Paris, FRA; 2 Oral Medicine Service, Hôpital Pitié Salpêtrière, Assistance Publique Hôpitaux de Paris, Paris, FRA; 3 UMR 8045 BABEL, Centre National de la Recherche Scientifique, Université Paris Cité, Institut Médico-Légal, Paris, FRA; 4 Oral Medicine Service, Hôpital Rothschild, Assistance Publique Hôpitaux de Paris, Paris, FRA

**Keywords:** preventive treatment, mandibular fracture, dentigerous cyst, osteosynthesis, patient-specific implants

## Abstract

Dentigerous cysts, if left untreated, can grow significantly and weaken the mandible, posing risks of pathological or iatrogenic fracture. Treatment options may include decompression, marsupialization, and enucleation, which are available, with the choice being multifactorial and tailored to each case. This article describes the management of two complex dentigerous cysts at risk of fracture, one about an 84-year-old woman and the second about a 41-year-old man. The cysts and associated teeth were surgically removed, and simultaneously custom-made titanium osteosynthesis plates were placed to prevent per- and postoperative fracture risks. This approach appears to be indicated in cases where marsupialization or decompression is impossible and when there is a high risk of iatrogenic fracture.

## Introduction

A dentigerous cyst, also known as a follicular cyst, is a benign odontogenic cyst, accounting for approximately 24% of all true cysts in the jaws. It is a developmental cyst, which develops due to fluid accumulation between the reduced enamel epithelium and unerupted tooth crown. The mandibular third molar is the most frequently involved tooth, followed by the maxillary canine and mandibular premolars [1].

Dentigerous cysts progress slowly and are usually asymptomatic, but they can become large and result in a palpable mass. In addition, as they grow, they can displace, resorb adjacent teeth, and weaken the jaws. Treatment options typically include enucleation, decompression, or marsupialization, with the choice depending on factors, such as cyst size, location, the need for unerupted tooth removal, and follow-up considerations [2].

Pathological mandibular fractures are rare, accounting for fewer than 2% of all mandibular fractures [3]. The mandibular angle and body are common locations for these fractures, which typically occur in areas where the bone has been weakened by an underlying pathological condition. They can result from various factors, such as cystic lesions [4,5], benign or malignant tumors, or metastatic disease, either spontaneously or due to external trauma. They can particularly occur during complex avulsions, such as third molars.

Several meta-analyses suggest that the presence of the third molar increases the likelihood of an angle fracture [6] by more than three times [7,8], particularly in classes C, II, and III according to the Pell and Gregory classification [8]. This classification determines the degree of impaction of the third molar in both vertical and horizontal dimensions [9], with the highest risk observed when third molars are classified as IIIC [7]. It has been demonstrated that the placement of a preventive plate effectively reduces stress on the mandible after the surgical removal of a cyst involving a third molar, based on a three-dimensional finite element (FE) analysis [10].

Advancements in computer-aided design and manufacturing (CAD/CAM) technology have made personalized medicine increasingly applicable in oral and maxillofacial surgery, enhancing treatment outcomes. The affordability and accessibility of this technology have improved, enabling the use of patient-specific implants in various procedures, such as temporomandibular joint (TMJ) total joint replacement, maxillofacial skeleton reconstruction, and orthognathic surgery [9].

The aim of this article is to report two cases and discuss the management of complex extractions associated with a dentigerous cyst using a patient-specific titanium plate to prevent iatrogenic mandibular fractures.

## Case presentation

Case 1

An 84-year-old woman was referred by her dentist for evaluation of a mandibular lesion that had been growing since 2014. Her medical history included high blood pressure, macular degeneration, and treated glaucoma. She also had reduced mobility and walked with the help of a crutch.

The lesion had been evolving chronically, with sporadic painful inflammatory episodes prompting her consultation. Clinically, there was a painful buccal swelling next to teeth 43 and 44, accompanied by a chronic fistula. Root canal treatments were performed on teeth 43 and 44 in 2022. Teeth 42, 41, 31, and 32 were vital but exhibited high mobility.

**Figure 1:**
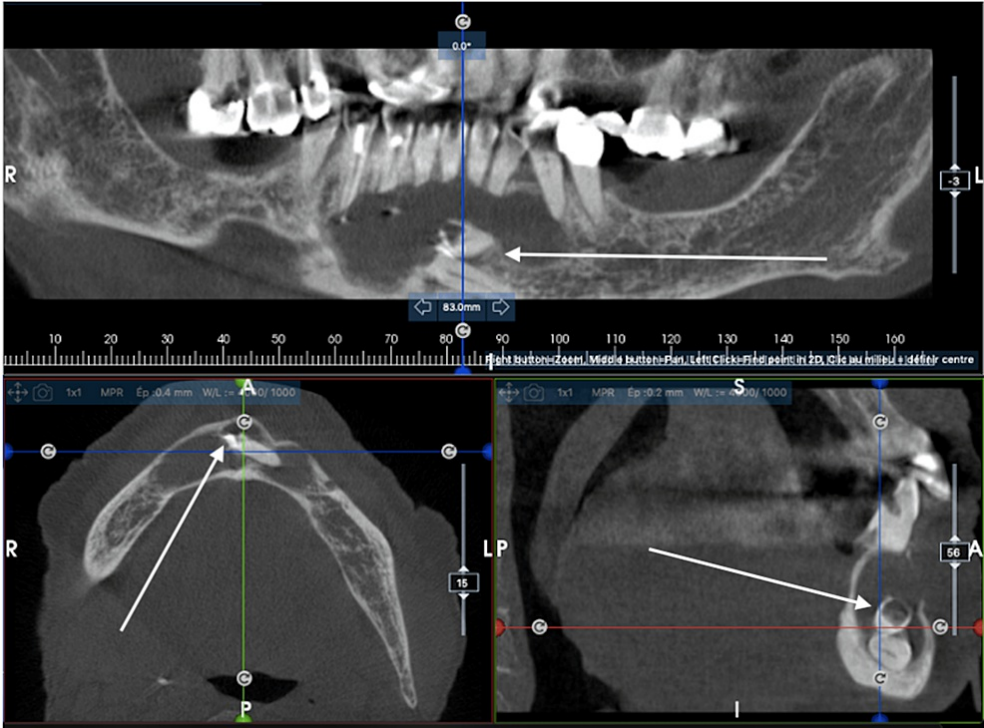
CBCT of case 1 showing the cyst and the impacted teeth. White arrows show the cyst with the two impacted teeth in the mandibular symphysis.

The lesion's size had tripled over 10 years, as evidenced by orthopantomograms from 2014 and 2023. Cone beam computed tomography (CBCT) (Figure 1) revealed a 30 mm-long axis lesion in the symphyseal region, which was well-demarcated and radiolucent, affecting the cortical bone and, extending from teeth 44 to 34. Two impacted teeth were identified within the cyst: tooth 33 and a supernumerary premolar 34 bis, both in contact with the basal bone of the symphysis. In addition, the cyst caused root resorption in teeth 44 to 32.

The clinical differential diagnosis included periapical cyst, odontogenic keratocyst, and ameloblastoma. However, a dentigerous cyst was the primary diagnosis, as the radiograph revealed unilocular radiolucency surrounding the cervical area of an unerupted tooth, with diffuse and thin corticated borders, characteristic features of a dentigerous cyst.

**Figure 2:**
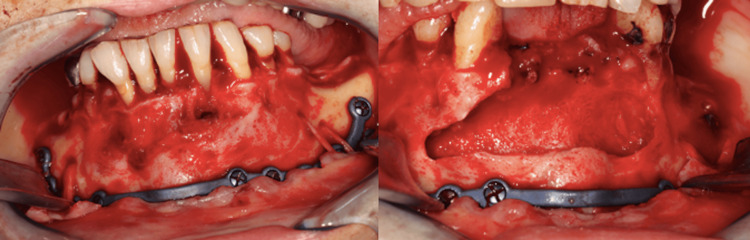
Clinical view of case 1 after osteosynthesis and cyst removal.

A complete enucleation of the cyst was planned, along with the extraction of teeth 41, 42, 31, 32, and 33. To reduce the risk of fracture during and after the surgery, a personalized titanium plate was fixed. The procedure was conducted under general anesthesia through an intraoral approach. The lower tooth was left in place due to the significant risk of fracture, absence of mobility, and lack of infection or residual cyst membrane (Figure 2).

Histological analysis confirmed that the cyst exhibited a squamous lining with nonspecific acute, subacute, and chronic inflammatory changes. No signs of malignancy, specific inflammation, or keratotic maturation were observed.

Case 2

**Figure 3:**
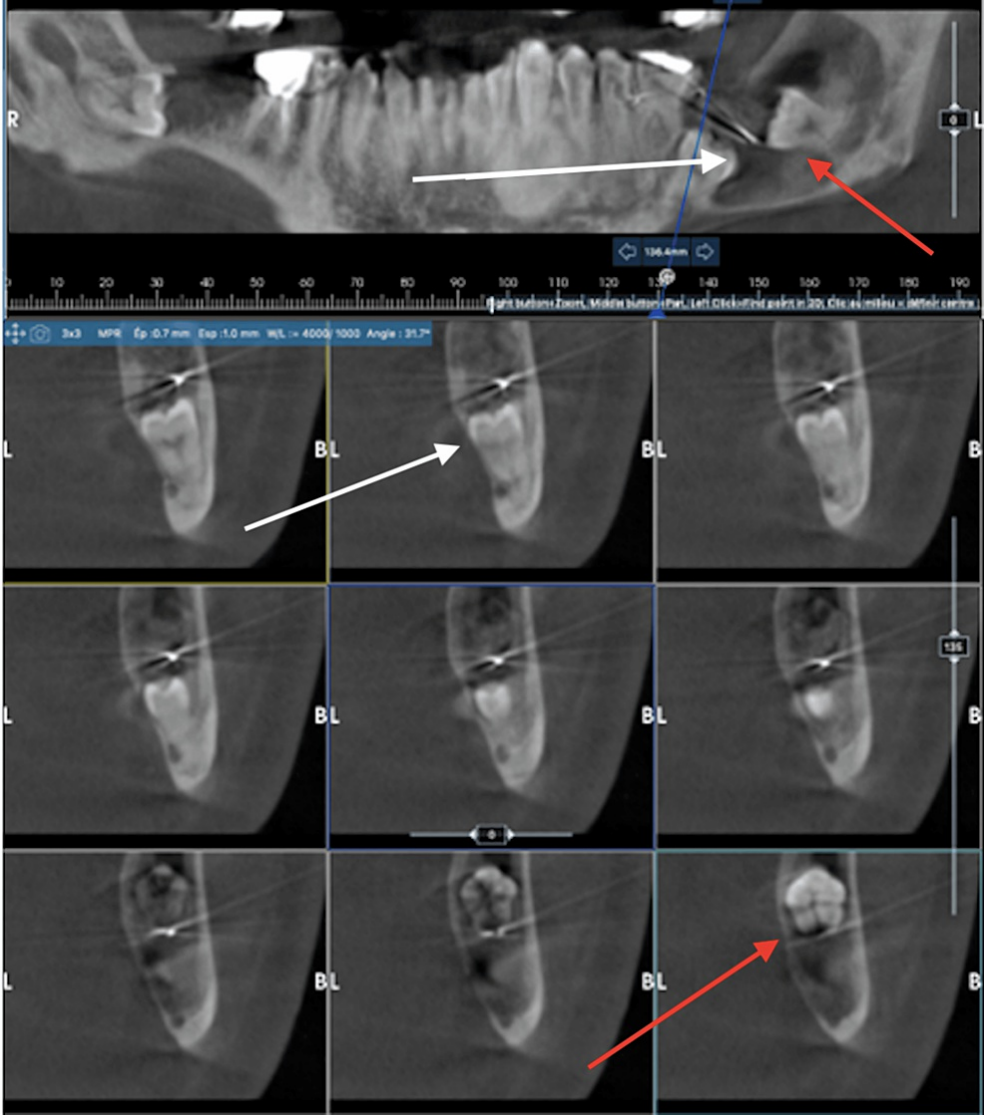
CBCT of case 2 showing the cyst and the impacted teeth. The white and red arrows show teeth 37 and 38 impacted in the cyst, respectively.

A 41-year-old man, in good health, was referred in June 2022 due to a mandibular angle cyst associated with impacted teeth 37 and 38. Endobuccal palpation revealed a resilient swelling, extending from tooth 36 along the ascending ramus. Clinical tests for teeth 34, 35, and 36 were normal. The panoramic radiograph showed impacted teeth 37 and 38, displaced to the basilar bone by a radiolucent lesion attached to the cervical area of tooth 38. Tooth 38 was classified as CIII according to the Pell and Gregory classification. The CBCT revealed a lesion measuring 50 mm at its largest axis, eroding the cortical bone in all three spatial dimensions. The distal root of tooth 36 was resorbed by tooth 37 (Figure 3). A biopsy confirmed the diagnosis of a remodeled dentigerous cyst.

**Figure 4:**
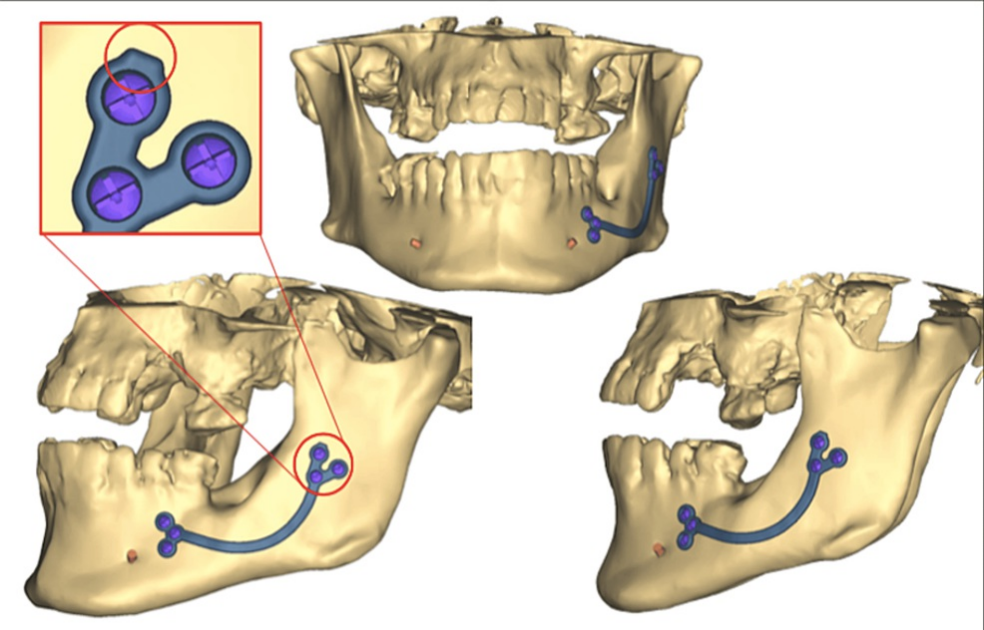
3D planning of the customed plate for case 2

Decompression was initiated on June 28, 2022, and continued for six months. The six-month follow-up CBCT showed a reduction in the size of the lesion, but there remained a persistent risk of fracture. Enucleation of the cyst and extraction of teeth 37 and 38 were performed under general anesthesia. A customized preventive osteosynthesis plate was placed using a drill guide with a transjugal approach (Figure 4). Histopathologic examination confirmed that the lesion was a remodeled dentigerous cyst.

A liquid diet for at least three weeks, followed by a soft diet for an additional three weeks, was prescribed for patients during the postoperative period. Follow-up appointments were scheduled for two weeks and one month post-surgery for routine evaluation. Clinical and radiological monitoring is essential to detect any signs of recurrence or complications.

## Discussion

Dentigerous cysts can be managed with several treatment options, including decompression, marsupialization, and complete enucleation. Marsupialization involves converting a cyst into a pouch by suturing the cyst lining to the oral mucosa. Similarly, decompression uses a drain to maintain communication between the cyst and the oral cavity, allowing for irrigation. These conservative methods are preferred, when preserving displaced teeth is desirable, especially in young patients, or when the cyst is large, posing a risk of surrounding tissue destruction and a pathologic fracture of the mandible [1]. Several cases of pathological mandibular fractures have been reported following third molar surgery or odontogenic cysts, including follicular cysts [4,11-13].

The disadvantage of marsupialization and decompression is the retention of pathological tissue in situ. Ameloblastoma, squamous cell carcinoma, or intraosseous mucoepidermoid carcinoma can develop from cells in the lining of a dentigerous cyst [14]. In addition, these methods require daily irrigation of the cystic cavity and long-term follow-up, sometimes up to two years for larger lesions [15]. In the event of device rupture or detachment, prompt replacement is necessary. Regular appointments are also required to adjust the device within the cystic cavity.

In the two cases presented, management decisions considered the patient's mobility issues and the risk of fracture. The first patient, with reduced mobility, was at risk of falls and pathological fractures. Her limited dexterity made it difficult to perform the necessary daily lavages for several months. The lesion was infected and associated with non-preservable teeth, justifying their extraction and cyst cleaning to prevent worsening and infection spread.

In both cases, the placement of a plate was considered to reduce the risk of perioperative and postoperative fractures. The risk was related to the weakening of the mandible due to the size of the lesion and the position of the teeth. The use of miniplates to treat mandibular fractures is well-documented in the international literature [16]. However, only two cases report using plates to prevent mandibular fractures during surgical extractions: one manually conformed [17] and one using a customized plate [18]. It appears that patient-specific plates are preferred over conventional mini plates for mandibular body fractures as they provide more stability, higher biting forces, and shorter operating times with highly acceptable outcomes and promising results [19].

Close communication between the laboratory and the surgeon allows for the design and positioning of the strongest possible plate and the future placement of osteosynthesis screws. Customized plates are created by laser fusion of titanium powder based on the 3D model of the mandible, matching the patient’s exact anatomy. Rigid and positioned according to the digital plan, these plates ensure passive preventive osteosynthesis, taking into account anatomical risks and post-surgical defects. This precise fit enhances stability and support, reducing the risk of displacement or failure. Customized plates enable faster and smoother surgical procedures with fewer complications or the need for revisions during and after surgery.

The disadvantages of this procedure include the number of additional steps and its cost. The overall process involves several phases: acquisition of axial slices in DICOM format (cone beam or CT scan), segmentation of anatomical structures, assessment of fracture risk on the 3D image, positioning of osteosynthesis screws by the surgeon, plate design by the biomedical engineer, project validation by the surgeon, manufacturing, sterilization, and finally the surgical procedure.

With additive manufacturing techniques gaining traction in medical fields, production costs are expected to decrease. Consequently, patient-specific osteosynthesis plates, designed to enhance stability, may see widespread adoption in the future [20].

## Conclusions

The size and location of the cyst, patient age, personal history, feasibility of regular follow-up, affected dentition, and involvement of critical structures are essential criteria for determining the treatment modality of a dentigerous cyst. Although the surgeon must consider the various industrial stages and additional costs, preventive custom-made mandibular plates are recommended in high-fracture-risk situations. This new approach, which to our knowledge has not yet been reported in the literature, appears to be particularly effective and suitable based on our initial feedback. It addresses the problem efficiently, offers ease of operational manipulation, and enhances security and predictability.
